# Supplementary material for: Finding the Appropriate Therapeutic Strategy in Patients with Neuroendocrine Tumors of the Pancreas: Guideline Recommendations Meet the Clinical Reality
Source: J Clin Med. 2021 Jul 7;10(14):3023. doi: 10.3390/jcm10143023 (PMC8304907; doi:10.3390/jcm10143023)
Supplement: Supplementary file 1 [file jcm-10-03023-s001.zip › jcm-1262063-supplementary.pdf]

Supplementary Materials:

The survey is attached as supplemental file. We also provide the basis of our statistical assessment as shown in supplemental table S1.

Supplemental Table S1: Summary of the case reports.

Case	Grading	Ki-67	Therapy line	Comment
C1	G2	<5%	2nd	PD under 1st-line SSA, comorbidities
C2	G2	<10%	1st	Asymptomatic, not resectable liver metastases
C3	G2	8%	2nd	PD under SSA after 12 months
C4	G2	<10%	3rd	PD after 10 months CTx (last 4 months CTx-free), prior SSA
C5	G1	1%	2nd	PD under 1st-line SSA for 3 years
C6	G2	15%	1st	Tumor-specific symptoms
C7	G2	15%	2nd	PD under 1st-line CTx, primary resected
C8	G2	20%	1st	Liver only metastasized, primary tumor in the tail of the pancreas
C9	G2	5%	3rd	PD under PRRT (4 cycles) after 1 year, prior SSA
C10	G1	1%	curative	3cm in size, no growth dynamic, no metastasis
C11	G2	5%	curative	3cm pancreatic primary; single liver metastasis (4 cm)
C12	G2	20%	2nd	PD under CTx, tumor specific symptoms, liver only disease
C13	G3	35%	2nd	CTx not tolerated and PD after 8 weeks
C14	G2	15%	1st	Declines CTx, elevated liver enzymes

PD, progressive disease; CTx, chemotherapy; PRRT, peptide receptor radionuclide therapy; SSA, somatostatin analogues.

Supplemental Table S2: Subgroup analysis based on the different disciplines participating.

	G1/2 Ki-67 <10%												G2 Ki-67 >10%										G3	
	C2		C1		C3		C5		C4		C9		C6		C8		C14		C7		C12		C13	
	n	%	n	%	n	%	n	%	n	%	n	%	n	%	n	%	n	%	n	%	n	%	n	%
**Endocrinology (n=18)**																								
SSA	13	72%					3	17%					1	6%	1	6%	3	17%	1	6%				
PRRT	1	6%	13	72%	9	50%	10	56%	10	56%			2	11%	1	6%	11	61%	9	50%	8	44%	7	39%
Chemotherapy	1	6%	4	22%	8	44%			1	6%	5	28%	11	61%	9	50%			5	28%	3	17%	6	33%
TKI			1	6%	1	6%			2	11%	8	44%							1	6%				
TACE/SIRT							3	17%	4	22%											2	11%		
Resection															3	17%								
OTH	3	17%					1	6%	1	6%	1	6%	2	11%	1	6%					1	6%	1	6%
**Gastroenterology (n=13)**																								
SSA	10	77%			1	8%													1	8%				
PRRT			6	46%	2	15%	6	46%	2	15%	1	8%	1	8%	1	8%	8	62%	6	46%	4	31%	3	23%
Chemotherapy	1	8%	2	15%	7	54%	1	8%	8	62%	7	54%	11	85%	9	69%			4	31%	4	31%	9	69%
TKI			2	15%	2	15%	1	8%	1	8%	4	31%					4	31%	1	8%				
TACE/SIRT	1	8%					4	31%	1	8%											3	23%		
Resection															2	15%								
OTH	1	8%	3	23%	1	8%	1	8%	1	8%	1	8%	1	8%	1	8%	1	8%	1	8%	2	15%	1	8%
**Nuclear medicine (n=20)**																								
SSA	14	70%					3	15%					3	15%	1	5%	1	5%						
PRRT	1	5%	16	80%	13	65%	12	60%	13	65%	1	5%	1	5%	3	15%	15	75%	13	65%	3	15%	10	50%
Chemotherapy	1	5%	2	10%	5	25%			1	5%	3	15%	9	45%	7	35%			1	5%	3	15%	7	35%
TKI			1	5%	1	5%			2	10%	12	60%	1	5%			1	5%	3	15%				
TACE/SIRT							4	20%	2	10%											10	50%		
Resection													2	10%	5	25%								
OTH	4	20%	1	5%	1	5%	1	5%	2	10%	2	10%	3	15%	2	10%			1	5%	1	5%		
**Oncology (n=16)**																								
SSA	16	100%					1	6%					1	6%	1	6%	3	19%						
PRRT			8	50%	6	38%	11	69%	4	25%			1	6%			11	69%	7	44%	6	38%	8	50%
Chemotherapy			2	13%	3	19%			9	56%	5	31%	11	69%	14	88%			5	31%	7	44%	7	44%
TKI			5	31%	5	31%	1	6%	2	13%	10	63%					1	6%	4	25%				
TACE/SIRT							2	13%													1	6%		
Resection													1	6%	1	6%								
OTH			1	6%	2	13%	1	6%	1	6%	1	6%	2	13%			1	6%			2	13%	1	6%
