# Supplementary material for: Finding the Appropriate Therapeutic Strategy in Patients with Neuroendocrine Tumors of the Pancreas: Guideline Recommendations Meet the Clinical Reality
Source: J Clin Med. 2021 Jul 7;10(14):3023. doi: 10.3390/jcm10143023 (PMC8304907; doi:10.3390/jcm10143023)
Supplement: Supplementary file 1 [file jcm-10-03023-s001.zip › Supplemental Material File S1 Complete presentation of the case scenarios..pdf]

1. In which medical setting do you work mainly?
- University Hospital
- not - university hospital
- Specialist practice
- Other

2. What is your clinical specialization?
- Oncology
- Gastroenterology
- Endocrinology
- Surgery
- Nuclear Medicine
- Internist
- Other

3. How many patients with NET do you treat on average per year?
- None at all
- 1 - 10
- 11 - 25
- 26 - 50
- More than 50
- Other

4. How much time of your work do you invest in the diagnosis and treatment of patients with NET?
- <5%
- 5-20%
- 21-40%
- >40%
- Other

5. A 65-year-old patient with a non-functional NET of the pancreas (G1/G2, Ki-67 <5%, and 68 Ga-PET/CT positive) that is inoperable due to comorbidities (ECOG 1-2) is undergoing treatment at your institution. What therapy would you recommend after documented tumor progression on somatostatin analogues?
- Chemotherapy
- Targeted therapy (Everolimus/Sunitinib)
- PRRT
- Other

6. A 75-year-old asymptomatic patient with NET of the pancreas and unresectable multiple bilobar metastases to the liver (G2, Ki-67 <10%) presents to you for initiation of therapy. Which therapy would you prefer?
- SSA
- Chemotherapy
- Targeted therapy (Everolimus/Sunitinib)
- Watch and wait
- PRRT
- Resection of the primary tumor and loco-regional treatment
- Other

7. A 70-year-old patient with unresectable NET of the pancreas (G2, Ki-67 8%, pulmonary and lymphnode metastases), received somatostatin analogues (SSA) for 12 months. Follow-up shows disease progression with a 30% increase in size according to RECIST criteria. What therapy would you recommend?
- Chemotherapy
- SSA

- Targeted therapy (Everolimus/Sunitinib)
- PRRT
- Other

8. A 70-year-old female patient with an unresectable NET of the pancreas and unresectable multiple metastases to the liver received SSA -therapy for 16 months followed by chemotherapy with STZ/5-FU. After 6 cycles, the disease was stable but showed tumor progression 4 months later without therapy. What therapy would you recommend?
    - Continue therapy with STZ/5-FU
    - Second-line chemotherapy with TEM/CAP or DTIC
    - Targeted therapy (Everolimus/Sunitinib)
    - PRRT
    - Loco-regional treatment (TACE/SIRT)
    - Other

9. An 80-year-old female patient withan unresectable NET of the pancreas (G1, Ki-67 1%) is treated with somatostatin analogues. The disease has been stable for 3 years under treatment. Now Ga68 PET/CT shows new liver metastases. What therapy would you recommend?
    - PRRT
    - Continue SSA
    - Targeted therapy (Everolimus/Sunitinib)
    - Loco-regional treatment (TACE/SIRT)
    - Other

10. A 75-year-old patient with a NET of the pancreas with progressive growth dynamics and symptomatic metastases (G2, Ki-67 15%) presents for consultation. What therapy would you recommend?
    - PRRT
    - Chemotherapy
    - Targeted therapy (Everolimus/Sunitinib)
    - Palliative resection
    - SSA
    - Other

11. A 78-year-old female patient after primary resection of pancreatic NET with additional hepatic and pulmonary metastases (G2, Ki-67 15%) has tumor progression after primary chemotherapy with temozolomide and capecitabine. What therapy would you recommend?
    - Surgical approach
    - Chemotherapy with DTIC
    - Targeted therapy (Everolimus/Sunitinib)
    - PRRT
    - SSA
    - Chemotherapy with STZ/5-FU
    - Other

12. A 65-year-old female patient with a NET of the pancreas (primary tumor in the tail of the pancreas) and multiple bilobar metastases to the liver (G2, Ki-67 20%) presents to you for a second opinion. What therapy would you recommend?
    - SSA
    - Chemotherapy with TEM/CAP or STZ/5-FU
    - Targeted therapy (Everolimus/Sunitinib)
    - PRRT
    - Watch and wait

- Surgical approach in combination with systemic treatment
- Other

13. A 72-year-old patient with a PanNET initially (G2, Ki-67 5%) received SSA therapy, during which the tumor was progressive. At the patient's request, PRRT was subsequently performed (4 cycles). However, tumor progression with new metastases occurred after one year. What therapy would you recommend?
- Chemotherapy with TEM/CAP or STZ/5-FU
- Targeted therapy (Everolimus/Sunitinib)
- Continue PRRT
- Other

14. A 70-year-old patient with a pancreatic NET (G1, Ki-67 1%) of 3cm size (localization in the pancreatic head) is under your treatment. The tumor shows no growth dynamic in imaging controls over 6 months. Ga68 PET/CT has ruled out metastasis. What therapy would you recommend?
- SSA
- Surgery
- Watch and wait
- Other

15. A 40-year-old female patient with pancreatic NET (G2, Ki-67 5%, primary tumor 32mm in size) shows an isolated liver metastasis of 40mm in segment VII in further staging. What therapy would you recommend?
- Resection of the primary and the liver metastasis
- Watch and wait
- SSA
- Chemotherapy with STZ/5-FU or TEM/CAP
- Neoadjuvant concept with PRRT and surgery
- Other

16. A 55-year-old patient with a hepatic metastatic pancreatic NET (G2, Ki-67 20%) presents progressive after 12 months of streptozotocin-based chemotherapy. The primary tumor was initially removed (Whipple procedure). Hepatic tumor burden is 25% and is bilobar. Tumor-specific symptoms are present. SSTR status is positive. What therapy would you recommend?
- Chemotherapy with TEM/CAP
- PRRT
- Chemotherapy with DTIC
- Loco-regional treatment (TACE/SIRT)
- Chemotherapy with carboplatin and etoposide
- Chemotherapy with STZ/5-FU
- Other

17. A 60-year-old patient with NET of the pancreas (G3, Ki-67 35%, hepatic and bone metastases) is being treated by you. The initiated therapy with temozolomide and capecitabine was discontinued due to therapy-specific side effects and at the patient's request. Short-term staging after 8 weeks shows tumor progression. What therapy would you recommend?
- Initiation of platinum-based chemotherapy
- Chemotherapy with STZ/5-FU
- PRRT (if Ga68 PET/CT is positive)
- Other

18. A 60-year-old female patient with a pancreatic NET (G2 Ki-67 15%, with lymphogenic and hepatic diffuse metastases) refuses classical cytotoxic

chemotherapy. There are 2-fold elevated transaminases. Ga68 PET/CT shows positive SSTR expression. What therapy would you recommend?

- SSA
- PRRT
- Loco-regional treatment (TACE/SIRT)
- Targeted therapy (Everolimus/Sunitinib)
- Other
